# A shape control method for soap bubble simulation using external forces

**DOI:** 10.1038/s41598-025-90850-2

**Published:** 2025-03-06

**Authors:** Arisa Horimoto, Syuhei Sato, Kaisei Sakurai, Keiko Nakamoto

**Affiliations:** 1 Faculty of Computer and Information Sciences, Hosei University, Tokyo, 184-8584 Japan; 2 Prometech CG Research, Tokyo, 113-0033 Japan; 3 CyberAgent, Inc., Tokyo, 150-0042 Japan

**Keywords:** Soap bubble simulation, Shape control, Target-driven control, Driving force, Computational science, Information technology, Software, Fluid dynamics

## Abstract

Simulating realistic behaviors of soap films is a challenging problem because the soap films have complex behaviors. In the computer simulation, various methods have been proposed to simulate these realistic behaviors. On the other hand, in computer graphics, many control methods have been proposed for fluid simulations, in order to create desired fluid animations for entertainment applications such as movies and video games. However, most of these studies do not focus on soap film simulations. This paper proposes a control method for soap film simulations in order to create soap bubbles with various shapes. Our control is performed by adding external forces to the simulation, and these external forces are calculated from user-specified target shapes. We adopt a surface-only soap film simulation based on the hyperbolic mean curvature flow because this formulation allows us to simply add external forces to motions of films. To enhance controllability around areas with sharp points, surface tensions are averaged for whole surface. Our system can also control magnitudes of global vibration until soap bubbles form target shapes by introducing intermediate shapes representing between an initial bubble and target shapes. We show control capability of our system by demonstrating various examples.

## Introduction

In computer graphics, physically-based simulations are often used to realistically represent natural phenomena. Especially, many methods have been proposed for simulating fluids such as water, smoke, fire, and so on. In this study, we focus on a soap film and bubble simulation among the fluid phenomena. Soap films and bubbles are curved surface with nature of the fluid. In order to represent these complex behaviours, various approaches have been proposed including mathematical ones^1–3^. Using these methods, realistic behaviours of soap films and bubbles can be represented. However, controlling these simulations as the user intended is difficult, users need to repeat trial-and-error processes for tuning parameters. On the other hand, many control methods have been proposed for fluid simulations^4–6^, in order to create desired fluid animations for entertainment applications such as movies and video games. However, these studies do not focus on soap film and bubble simulations.

The goal of our research is to represent soap bubbles with various shapes by controlling a simulation. In this paper, we adopt a soap bubble simulation proposed by Ishida et al.^1^, and extend the external force-based smoke control method^4^ for controlling the shapes of the soap bubbles. Our system calculates external forces (driving forces) directed to a target shape specified by users. The target shape is given as a 3D mesh model and transformed to a field representing only around its outline because motions of bubbles are computed only for its surface. Then, the driving forces are calculated from the outline field and added to vertices which discretize the surface of bubbles. However, in regions with sharp points, the surface tension can be locally significant, leading to cases where the soap bubble cannot be controlled according to the target shape. To address this, we average surface tensions for all the vertices of the bubble surfaces. Our system can also control magnitudes of global vibrations of soap bubbles by introducing intermediate shapes representing between an initial shape and a target shape of soap bubbles. We demonstrate several examples to show usefulness of our method.

The primary focus of shape control research in computer graphics has been on phenomena such as smoke (grids) and liquid (particles). However, there has been no prior research on shape control for surface-only simulations, like soap bubbles, which are strongly influenced by surface tension that drives them back to a spherical form. Our method, while an extension of classical external force-based control, is the first to focus on shape control for soap bubble simulations. Also, this approach represents an efficient method for solving inverse problems in shape modeling using simulations. This approach has the potential to serve as a foundation for the development of more efficient methodologies in the future. Additionally, the state-of-the-art fluid control methods predominantly rely on optimization-based approaches, which require computational costs on the order of several hours^7,8^. In contrast, our method adopts a potential-based approach^4^ and requires computational costs ranging from several tens of seconds to a few minutes, which are significantly lower than those of recent optimization-based techniques.

## Related work

### Soap bubble simulation

Visual simulation of films and bubbles has been a long-standing subject of study in computer graphics^9–14^. When simulating behaviors of soap films and bubbles on a computer, challenges arise due to the computational complexity and the difficulty in achieving visually appealing representations. Addressing these challenges, Ishida et al. discovered that the behavior of films is closely related to a geometric flow called hyperbolic mean curvature flow. They modified and formulated this hyperbolic mean curvature flow to be applicable for simulating films^1^. Ishida et al. demonstrated that this formulation is equivalent to a simplified form of the Navier–Stokes equations commonly used in traditional fluid simulations. This modification enabled faster, more robust, and visually stunning simulations compared to conventional methods. Furthermore, Ishida et al. derived a set of motion equations by introducing the thickness of the film as a degree of freedom into the Navier–Stokes equations. This allowed for simulations of bubbles that consider the thickness of the film^2^. In this simulation, additional effects, such as convection, undulation, drainage, and evaporation of the thin film, were introduced to extend the simulation of bubbles. Huang et al. developed a chemomechanical simulation framework based on lubrication theory^15^. They customized the semi-Lagrangian advection solver, enabling the simulation of soap film dynamics on spherical bubbles under gravity or external airflow. Deng et al. proposed a novel mesh-free method called Moving Eulerian-Lagrangian Particles (MELP) for simulating incompressible fluid on the surface of thin films and bubbles^3^. MELP adopts a two-layer particle structure, allowing the calculation of intricate and dynamic flows and significant surface deformations with high stability and efficiency. Considering ease of introducing external forces, we utilize the soap bubble simulation proposed by Ishida et al.^1^.

### Discretizing surface tension forces

Surface tension is one of important factors for the simulation and control of soap films. Hyde et al. introduced an updated Lagrangian discretization of surface tension forces^16^. This approach works with Particle-In-Cell and Material Point Methods (MPM), and can simulate high surface tension materials, such as liquid metals. They designed discrete forces as the gradients of the potential energy, associated with surface tension, with respect to the motion of the fluid over a time step. Chen et al. presented an MPM discretization of surface tension forces arising from spatially varying surface energies^17^. This method can simulate materials with highly diverse degrees of surface tension and thermomechanical effects, such as water, wine, and wax. The discretization is based on surface energy, allowing this energy-based approach to automatically capture surface gradients without explicitly resolving them, as required in traction-condition-based approaches commonly used in particle method discretizations. In the soap bubble simulation we adopt^1^, surface tension is defined at each vertex of the discretized surface meshes. When controlling the bubble into sharp shapes, this surface tension can become extremely large, significantly affecting the control performance. To address this, we average the surface tension forces across the entire surface, improving control performance toward the target shape and suppressing unnecessary oscillations.

### Fluid control

Many control methods have been proposed for representing fluids with various shapes. Treuille et al. developed a smoke control method through user-specified keyframes^18^. This methods control the simulation using external forces obtained by solving a continuous quasi-Newton optimization. McNamara et al. controlled fluid simulations through gradient-based nonlinear optimization^19^. This method adopts the adjoint method for efficiently computing derivatives. Hong and Kim used control forces obtained from a potential field^20^. Fattal et al. introduced a driving force which is an external force fluids are directed to a target shape^4^. Shi et al. also used the driving force, and additionally introduced another external force which is computed according to differences between target shapes and fluids^5^. This method can control liquid motions to rapidly changing target shapes. Thürey et al. proposed locally defined external forces from control particles^6^. Because this method controls only low-frequency components of flows, detailed motions of fluids can be preserved. Dobashi et al. controlled simulations such that clouds reach to a user-specified curve which represents target heights^21^. Parameters related to cloud growth are automatically adjusted according to height differences between the target and the simulated cloud. Sato et al. introduced stream functions to deform existing simulated smoke flows^22^. Deforming smoke flows in stream function space, incompressibility condition is always satisfied for deformed flows. Sato et al. proposed a method for simply controlling a size and a flow direction of the fire^23^. In this method, external forces and a source temperature are automatically adjusted by feedback control according to differences between distributions of target and current fire temperatures. Guide-based methods also can control fluid flows. Forootaninia and Narain developed a guide simulation in frequency space^24^. This method is simple but effective for guiding smoke flows. Sato et al. guided smoke simulations in stream function space^25^. This method is robust to non-physical inputs generated procedurally, or handdrawn by the user. These methods focus mainly smoke, liquid, and fire, but no methods have yet been developed for controlling soap bubble simulations.

### Shape morphing

In the shape control of soap bubbles, bubble shapes gradually change from one to another, so it can be considered as shape morphing. Many researches have traditionally been conducted for the shape morphing^26–30^. Among those researches, physically-based morphing is most closely related to our objective. Min et al. presented a control framework for underwater soft-bodied animals^31^. This method simulates and controls muscle excitation based on a deep reinforcement learning algorithm. Xu et al. achieve physically-based shape morphing for elastic shapes^32^. This method can accommodate complex topology changes, leveraging the differentiable MPM and space-time control through perparticle deformation gradients. Shape interpolations can also achieve the shape morphing^33,34^. Raveendran et al. proposed an interpolation method between multiple liquid surfaces^35^. In this method, registration of meshes is done through a new space-time non-rigid iterative closest point algorithm. Thuerey also developed an approach for interpolating liquid and smoke animations^36^. This approach calculates a dense space-time deformation through grid-based signed-distance functions. Our method enables shape morphing through the simulation and control of soap bubbles. Additionally, by using the method proposed by Turk et al.^27^, intermediate states between multiple target shapes are generated, which helps control the magnitude of the bubble’s oscillations.

**Fig. 1:**
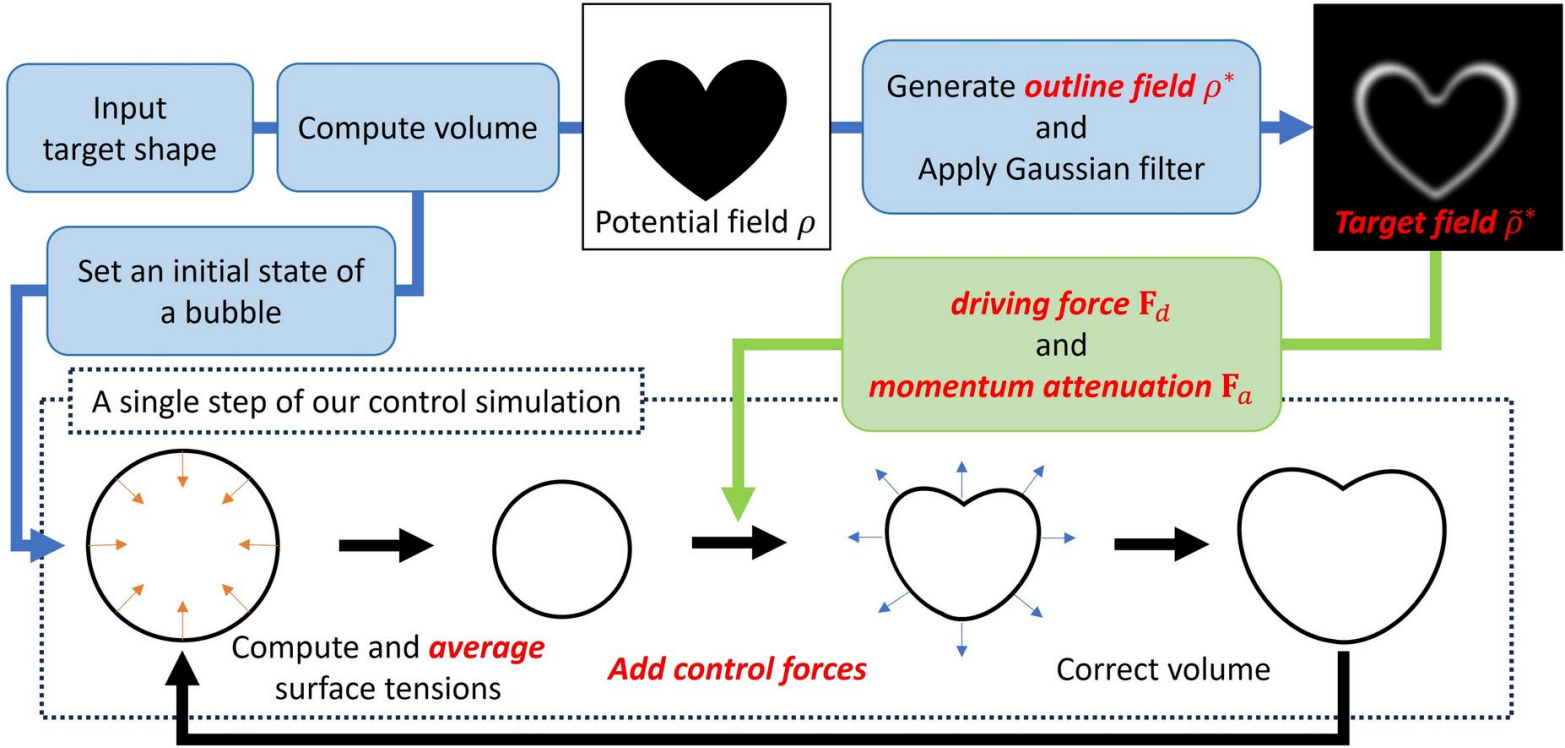
Overview of our control method. For visual clarity, the input shape, target field, and bubbles are illustrated in 2D.

## Soap bubble simulation

Ishida et al. discovered that the behavior of thin films is closely related to a geometric flow known as the hyperbolic mean curvature flow^1^. They then modified the hyperbolic mean curvature flow to enable volume preservation and demonstrated that this modification is equivalent to a simplified form of the Navier–Stokes equations in the soap film simulation.

Traditional soap bubble simulations have primarily been based on the Navier–Stokes equations. The Navier–Stokes equations consist of advection, pressure, viscous, and external force terms, with the version excluding the viscous term referred to as the Euler equations. Ishida’s formulation is shown to be derivable from the Euler equations considering surface tension, under the assumption that the film is extremely thin and the pressure is constant. In this method, a triangular mesh is used to discretize the film in space. The behavior of the film is computed by updating the positions of each vertex of the mesh. The update of each vertex follows the hyperbolic mean curvature flow defined by the following equation.

1 $$\begin{aligned} \frac{d^2 \textbf{x}}{dt^2} = - \beta H(\textbf{x}, t) \textbf{n}(\textbf{x}, t), \end{aligned}$$

where, $\textbf{x}$ is a position of each vertex, $\beta$ is a constant equals to twice the surface tension coefficient $\sigma$, *H* indicates mean curvature, and $\textbf{n}$ is a normal vector for each vertex. Equation (1) indicates that each vertex on bubble surfaces is accelerated directed to mean curvature normal. In Ishida’s formulation, to accommodate the hyperbolic mean curvature flow even at the junctions of soap bubbles where the mean curvature is indefinite, they utilize the gradient of the area of each surface $\partial A(\textbf{x})/\partial \textbf{x}$ instead of the mean curvature normals for calculating surface tension in soap bubbles. This gradient is known to be equivalent to the mean curvature normal on a smooth surface. Additionally, the volume inside the soap bubble is preserved by evolving the surface in the normal direction based on the difference in pressure between the interior and exterior of soap bubbles. The final model is as follows.

2 $$\begin{aligned} \frac{d^2 \textbf{x}}{dt^2}=-\beta \frac{\partial A(\textbf{x})}{\partial \textbf{x}}+\Delta p \textbf{n}. \end{aligned}$$

In this formulation, external force terms can simply be added to Eq. (2). Utilizing this property, we will achieve shape control by adding external forces which surfaces of soap bubbles are moved to the target shape. Note that the first term of the right hand side in Eq. (2) is equivalent to the right hand side in Eq. (1) because we treat only soap bubbles without junctions. Therefore, we compute surface tensions based on the mean curvature normal defined by the right hand side in Eq. (1).

## Our method

Figure 1 shows an overview of our control simulation. As a preprocess, we prepare a target field and a target volume based on a user-specified input shape (the upper row in Fig. 1). The input is given as 3D mesh models. First, a potential field $\rho$ defined on a 3D grid space is obtained from the input mesh. In this potential field, the grid located inside/outside the input mesh has a value of 1/0. The target field and the target volume are computed from this potential field $\rho$. The target volume is used for the volume preservation described in section “Soap bubble simulation”.

Then, in the runtime simulation, the shape control consists of the driving force and momentum attenuation is executed between the calculation of the surface tension and volume preserving (the lower row in Fig. 1). Additionally, surface tensions are averaged for all the vertices to avoid locally large surface tensions around regions where shapes of films are sharp. Our method can also control a magnitude of global vibrations until soap bubbles form into the target shape, by introducing several intermediate shapes which represent those between an initial bubble and target shapes. These intermediate shapes are used only for computing the target fields. On the other hand, the target volume is calculated from only the user-specified target shapes.

In our control method, multiple target shapes can be specified as keyframes. In this case, the target volume is calculated by alpha blending between volumes of each target shape, and intermediate shapes are calculated between each keyframe. In the following, we describe details of each process.

**Fig. 2:**
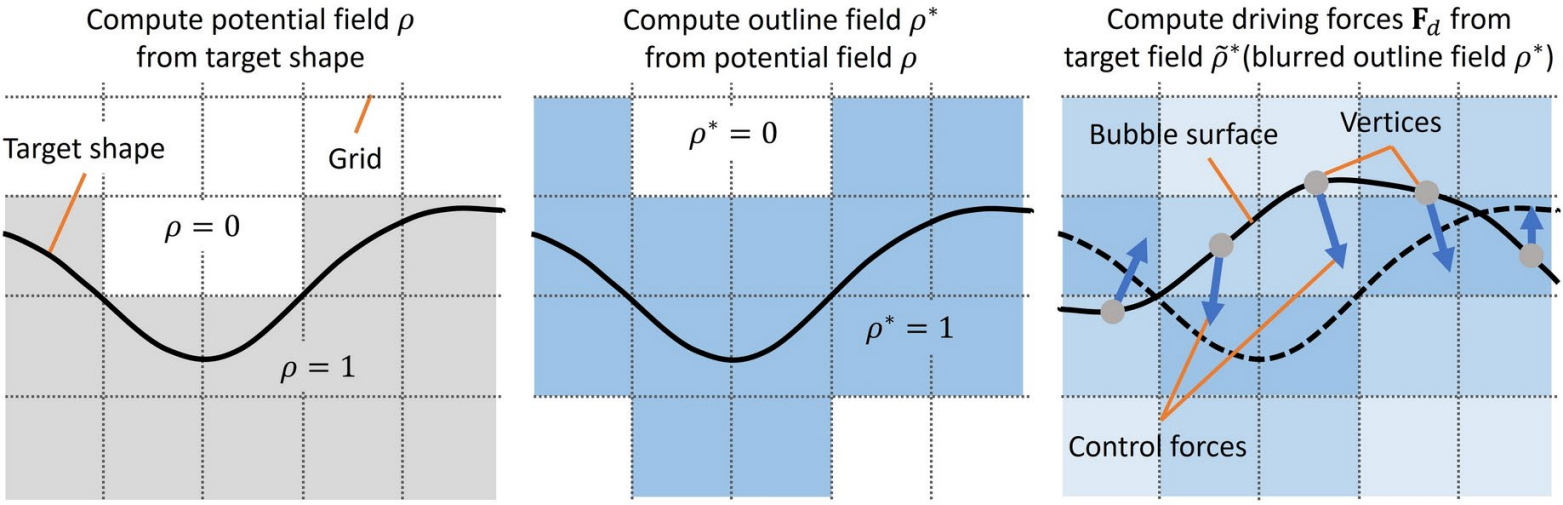
Overview of computing driving forces at each vertex on the bubble surface. For visual clarity, we illustrate this figure in 2D.

### Calculating target field

A target field is created based on the potential field $\rho$ obtained by the user-specified target shape. However, using the same target field as Fattal et al.^4^, the driving forces are directed to the inner region of the input shape. This is not suitable for our purpose: our objective is to control a bubble shape such that a surface of the bubble is directed to an outline of the input shape. Therefore, our system newly defines an outline field $\rho ^*$ from the potential field $\rho$ (Fig. 2): a grid value is 1 around the outline of the input shape and is 0 at the other regions. A width of the outline region is specified by users. Then, a Gaussian filter is applied to the outline field, and this filtered version of $\rho ^*$ is our target field $\tilde{\rho ^*}$ (Fig. 2 right). Driving forces are computed based on this target field $\tilde{\rho ^*}$.

Additionally, a target volume is also computed based on the potential field $\rho$: the total number of grid cells in the potential field that are located inside the target shape (cells with a value of 1) is counted, and this value is adjusted to the spatial scale to obtain the target volume.

**Fig. 3:**
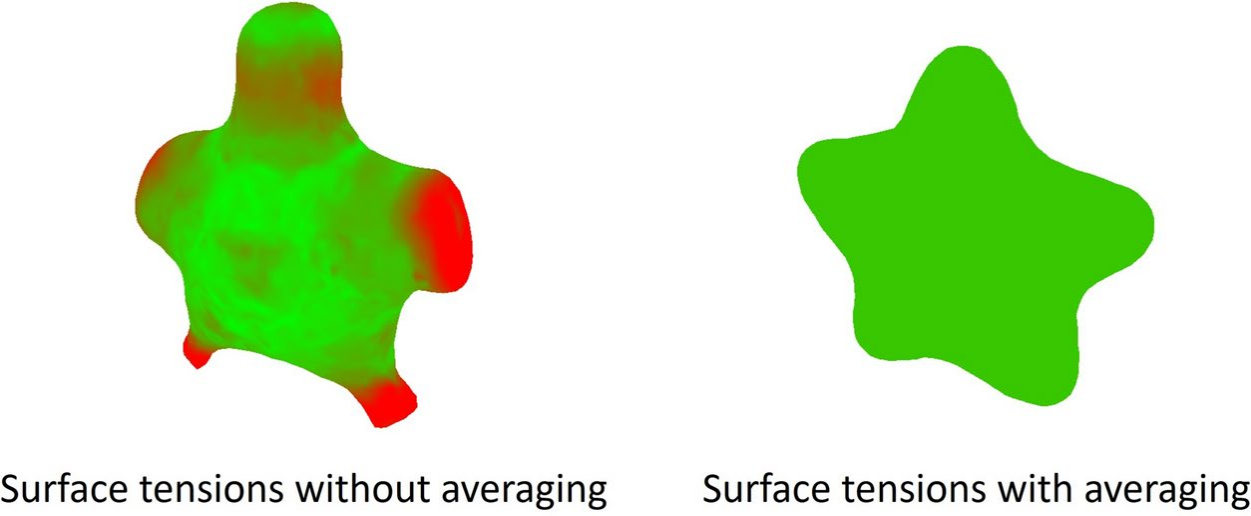
Effects of averaging surface tensions. Tensions are visualized using pseudo colors, where red represents the maximum magnitude of tension, and green represents zero tension.

### Adding control forces

Fattal et al. controlled the shape of smoke by introducing two additional external forces termed as driving force term and momentum attenuation term to the Navier–Stokes equations^4^. The driving force term applies forces to guide the smoke towards user-specified target shapes, while the momentum attenuation term restricts the accumulation of momentum to prevent the smoke from oscillating near the target shapes.

However, Fattal’s method targets grid-based fluid simulations. In grid-based approaches, the simulation space is divided into a grid, and the behavior of fluid is simulated by computing velocities and pressures at each grid point. Therefore, control forces are also computed at grid points. On the other hand, the behavior of the soap bubble is calculated at each vertex on its surface, thus not directly corresponding to grid points. Therefore, the forces acting on each vertex are linearly interpolated from the driving force field defined on the grid. We compute the driving force $\textbf{F}_d$ at each grid point from a target field $\tilde{\rho ^*}$ as follows, referenced Fattal’s one:

3 $$\begin{aligned} \textbf{F}_d = \nu _d \frac{\nabla \tilde{\rho ^*}}{\tilde{\rho ^*}}, \end{aligned}$$

where, $\nu _d$ is a user-specified non-negative coefficient to adjust the magnitude of the driving force. If this coefficient is too large, the soap bubble will deform excessively beyond the target shape. Conversely, if it is too small, the ability to control the bubble toward the target shape will be diminished. For the momentum attenuation term, we add the following external force $\textbf{F}_a$ (the same as the Fattal’s one) to each vertex:

4 $$\begin{aligned} \textbf{F}_a = -\nu _a \textbf{u}, \end{aligned}$$

where, $\textbf{u}$ is a velocity at each vertex. $\nu _a$ is a user-specified non-negative coefficient. If this coefficient is too large, it overly suppresses the movement of the soap bubble. Conversely, if it is too small, it fails to adequately restrain the large forces generated by driving forces or surface tension.

### Averaging surface tension

The driving force obtained from the spatial gradient of the target field deforms the soap bubble, while the momentum attenuation term reduces the force acting on the soap bubble over time, preventing the surface of the soap bubble from large oscillations near the target field. However, depending on the shape of the target, such as shapes with sharp points, there may be cases where the force exerted on the surface of the soap bubble by the surface tension term in Eq. (1) becomes biased, resulting in the soap bubble not being controlled into the target shape.

This occurs because in areas with high surface curvature, such as sharp points, both the driving force towards the target shape and the surface tension towards a stable shape become significant. However, reducing the driving force to address this issue would result in the loss of control towards the target shape. Additionally, we confirmed through preliminary experiments that the momentum attenuation term is not effective in suppressing such locally strong forces, and increasing $\nu _a$ does not improve control performance. Therefore, we average the surface tension at each vertex on the surface of the soap bubble based on the sum of their magnitudes each frame (Fig. 3). This approach allows us to maintain the unique movements of the soap bubble surface while still controlling it towards the target shape, even in regions with sharp points.

The surface of soap bubbles naturally forms spherical shape rather than flat shape in equilibrium, meaning the surface tension across the bubble is not zero but has a specific magnitude which is similar for all surfaces. Our averaging method takes inspiration from this phenomenon, aiming to suppress the significant increase in surface tension, particularly in sharp points.

### Introducing intermediate shapes

Introducing intermediate shapes transitioning from the initial state of the soap bubble to the target shape controls the magnitude of global oscillations of the soap bubble until it forms the desired shape. Since our control relies on external forces, there may be instances where the driving force is continuously added in the same direction in areas where the soap bubble differs significantly from the target shape, leading to large global oscillations of the soap bubble. We control the magnitude of these oscillations by introducing intermediate shapes and gradually transitioning them to the target shape.

We create intermediate shapes based on the method proposed by Turk and O’Brien^27^. First, grid-based signed distance functions are computed for both the initial state and target shapes. In these distance functions, grid points located on the surface of each shape have a value of 0, while the distance from the nearest surface is stored for all other grid points. Additionally, grid points inside the shapes have their distances multiplied by -1. Then, by alpha-blending these distance functions, we create several distance functions representing the intermediate shapes. Finally, we calculate the target fields from these distance functions. When users specify multiple target shapes, we compute intermediate shapes between each shape.

In our method, the magnitudes of global oscillation of the soap bubble until it forms the target shape can be controlled by adjusting either the number of intermediate shapes used ($N_I$) or the time intervals at which intermediate shapes are switched ($\Delta t_I$). Examples of varying these parameters are demonstrated in the following section.

**Fig. 4:**
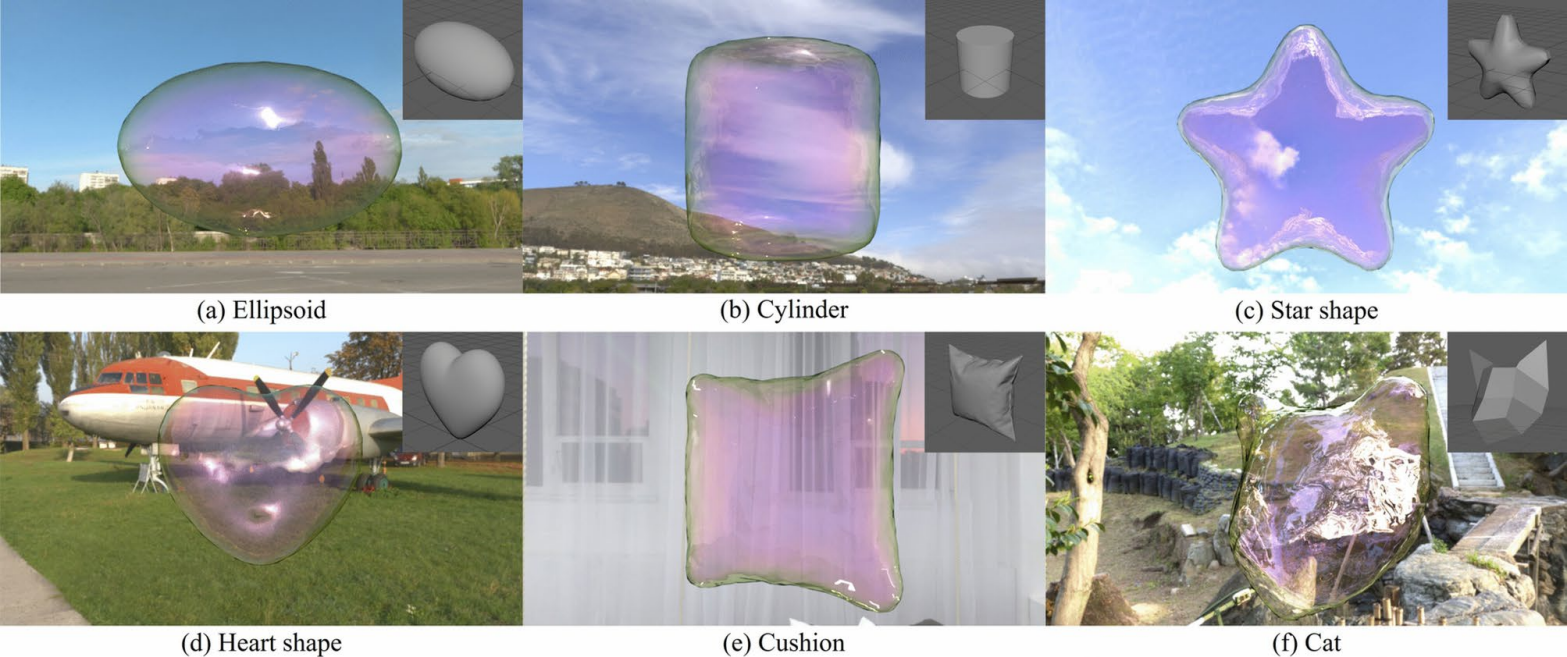
Our results with various target shapes. The inset image on top-right in each result indicates the target shape.

**Fig. 5:**
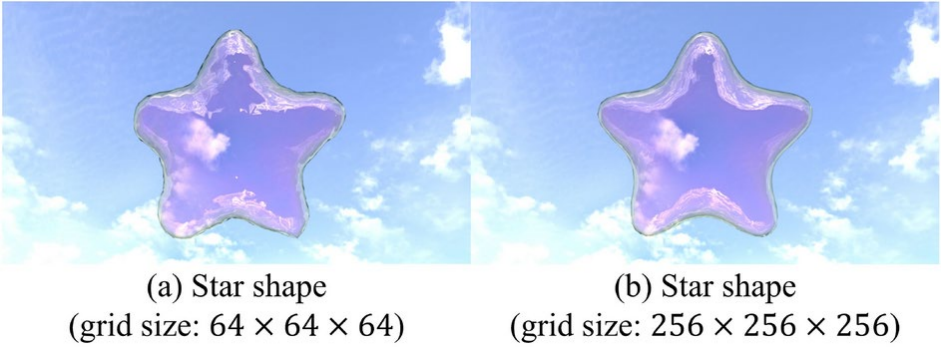
Results with different grid sizes.

**Fig. 6:**
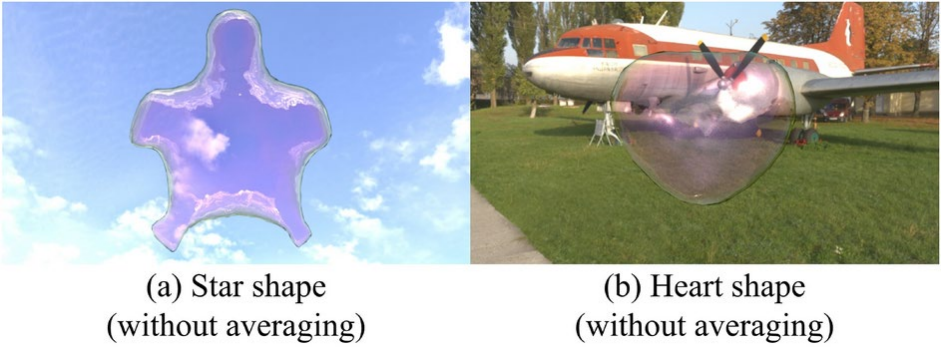
Results without our averaging.

**Fig. 7:**
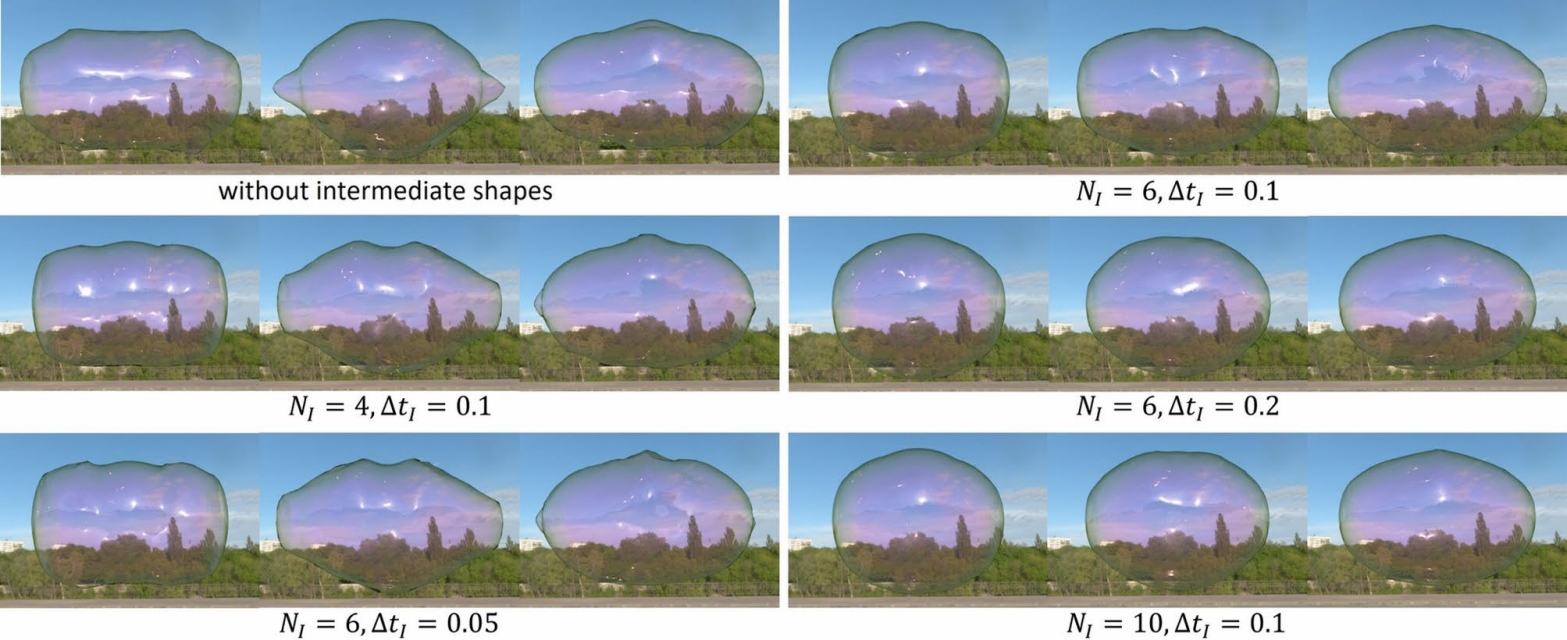
Comparison of our results with varying two parameters relating to intermediate shapes. $N_I$ is a number of intermediate shapes, $\Delta t_I$ is a time interval to switch each intermediate shape. The simulation time elapses from left to right.

**Fig. 8:**
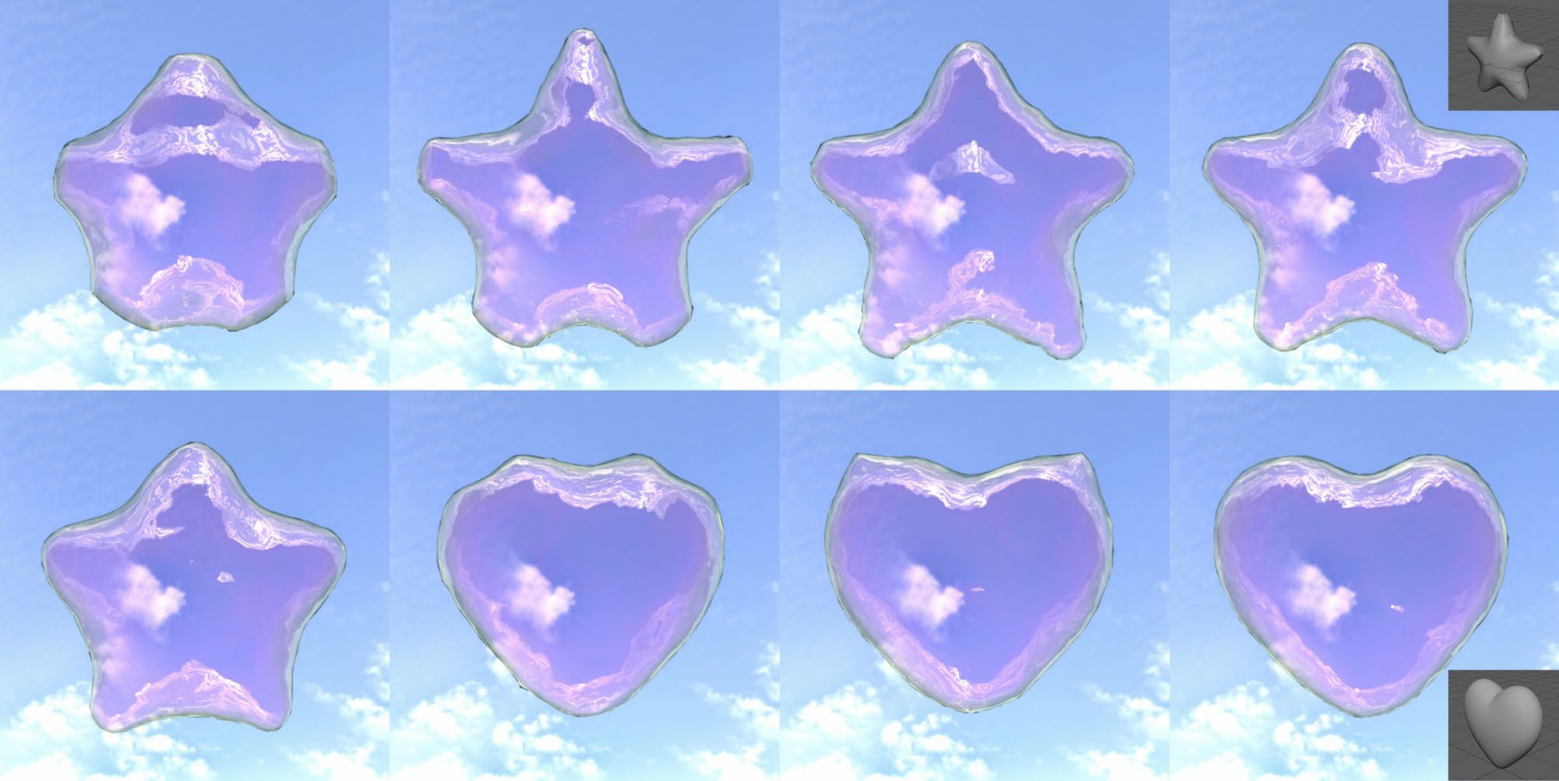
An example of multiple target shape. Star shape is set to the 1st target and heart shape is set to the 2nd target. In this example, intermediate shapes are used ($N_I = 6$, $\Delta t_I = 0.1$). The simulation time elapses from upper left to lower right.

## Results

Several examples are shown in Figs. 4, 5, 6, 7 and 8. We used a desktop PC with an Intel Core i9-13900K CPU and 64GB of memory to compute the examples. For all the examples, unless otherwise stated, a grid size of the target field is $128\times 128\times 128$, a window size of the Gaussian filter is $20\times 20\times 20$, and an initial state is set to a spherical shape with a volume the same as each target shape. Note that when the window size of the Gaussian filter is too small, the gradient of the target field becomes zero in regions far from the target shape. Table 1 summarizes the hyperparameter values, and the computation time required for simulation and control, for each result. Our control simulation is only about 25–30 percent slower than the computation time for the original soap bubble simulation^1^. Resultant soap bubbles are rendered using *Mitsuba renderer*^37^ extended to be able to represent structure colors. Animations of these examples are contained in the supplemental video.

**Table 1:** The average number of verticies and faces, the volume of the bubble, the time interval $\Delta t$, the surface tension coefficient $\sigma$, the hyperparameter ($\nu _d$, $\nu _a$) values , and the computation time for simulation ($T_s$) and control ($T_c$) [sec./frame], for each result.

Results	Verticies	Faces	Volume	$\Delta t$	$\sigma$	$\nu _d$	$\nu _a$	$T_s$	$T_c$
Figure 4a	2183	4362	0.659	0.01	1.0	15.0	0.3	0.290	0.073
Figure 4b	2518	5032	0.775	0.01	1.0	15.0	0.3	0.281	0.072
Figure 4c	2784	5564	0.735	0.01	1.0	12.0	0.3	0.283	0.077
Figure 4d	1405	2806	0.295	0.01	1.0	15.0	0.3	0.237	0.066
Figure 4e	3960	7916	1.375	0.01	1.0	15.0	0.3	0.223	0.060
Figure 4f	2447	4890	0.697	0.01	1.0	15.0	0.3	0.204	0.051
Figure 8	2674	5344	0.735	0.01	1.0	15.0	0.3	0.255	0.071

First, several examples created using our control are shown in Fig. 4. The target shape is shown in the top-right on each image. The bubble is successfully controlled into the target shape for each example. Especially, we found bubble surfaces can be controlled into the target shape also at the sharp edges in the star and heart shapes. However, at the sharp tips of certain shapes, small fragments of the soap bubble may tear off and separate before forming the target shape. This occurs due to a sudden increase in the control force, but it can be suppressed by introducing intermediate shapes. The following section will present experimental examples demonstrating this effect.

To validate the effectiveness of the proposed method, we compare the results based on differences in the grid resolution used to define the driving forces and the presence or absence of averaging, as shown in Figs. 5 and 6, respectively. Fig. 5 shows the results when the grid size from Fig. 4c is changed to $64\times 64\times 64$ (a), $256\times 256\times 256$ (b). In (a), the tips of the star appear slightly thicker than in Fig. 4c. However, no significant differences were observed across the grid resolutions. Figure 6 illustrates the results without applying averaging for a star shape (a) and a heart shape (b). In (a), the soap bubble undergoes significant deformation, and large oscillations persist over time, preventing the bubble from achieving the desired target shape. In (b), although the deformation is less severe than in (a), large oscillations still persist, and only partial control of the target shape is achieved. These results indicate that averaging significantly influences control performance, especially when the target shape contains many sharp features.

Next, we show a comparison of results when the two parameters $N_I$ and $\Delta t_I$ relating intermediate shapes are varied (Fig. 7). The images show several frames before the soap bubble is formed into the target shape. In all cases, the final shape is controlled to the one shown in Fig. 4a, but the oscillations during the control simulation are different depending on the value of the parameters. When $N_I$ or $\Delta t_I$ is small (the 2nd and 3rd row in Fig. 7), the oscillations are relatively large close to the one without the intermediate shapes (the 1st row in Fig. 7). In contrast to these results, when $N_I$ or $\Delta t_I$ is large, the oscillations can be suppressed. As shown in this comparison, by adjusting these parameters, we can control the shape of soap bubbles while also controlling the magnitudes of the oscillations.

Finally, we show an example of multiple target shapes (Fig. 8). Star shape (same as Fig. 4c) is set to the 1st target, and heart shape (same as Fig. 4d) is set to the 2nd target. In this example, intermediate shapes are prepared for each section, the initial state to the 1st target, and the 1st target to the 2nd target. The two parameters are set to the following: $N_I = 6$, $\Delta t_I = 0.1$. The surface of the soap bubble is successfully controlled also on the transformation from the star shape to the heart shape.

**Fig. 9:**
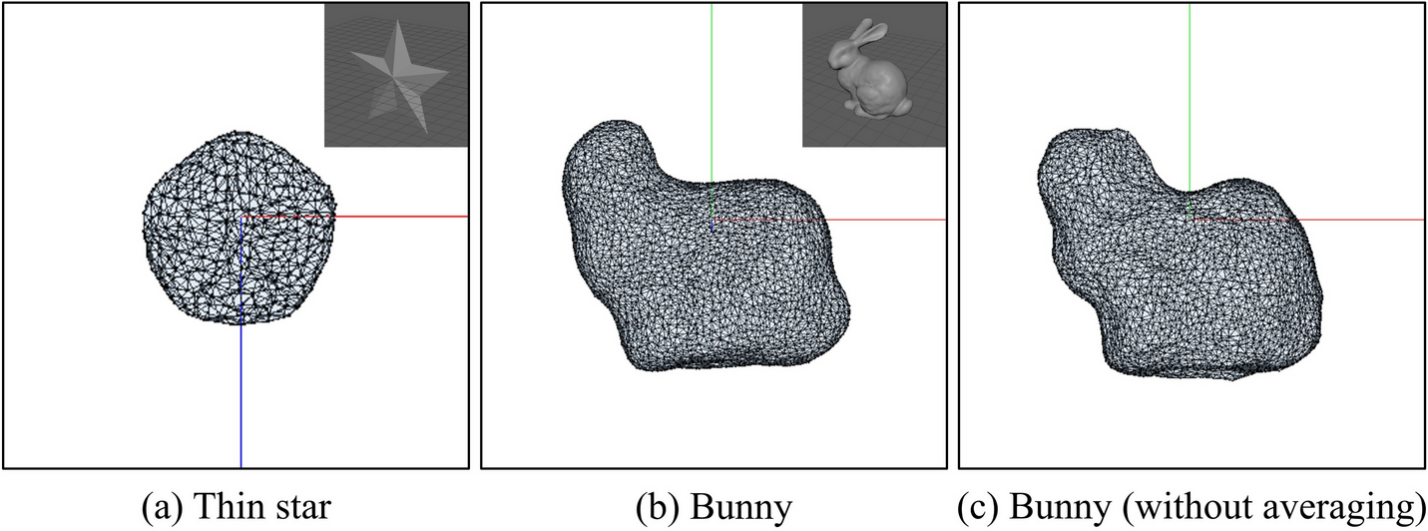
Failure cases of our control. Both results are rendered as the wire frame model. The inset image on the top-right on each result is the target shape.

## Limitation

Our method can control the shape of bubbles as shown in section “Results”. However, for some shapes, the soap bubble was not controlled according to them (Fig. 9). For instance, when a thin star shape was specified as the target shape, the resulting shape resembled a pentagon (Fig. 9a). When the Stanford Bunny was set as the target shape, the contour of the ears could not be accurately represented, resulting in only a rough approximation of the overall shape (Fig. 9b). The primary reason for the inability to control the soap bubble as intended was attributed to the surface tension of the soap bubble acting to form a spherical shape, which is the equilibrium state. Additionally, as shown in Fig. 9c, control is also not achieved when the averaging is not applied, and significant oscillations persist compared to the control with the averaging.

As one potential solution, controlling the shape using multiple soap bubbles could be considered. For example, in the case of the Stanford Bunny, it might be possible to achieve control closer to the target shape by using three soap bubbles: one for each ear and one for the rest of the body. However, this approach would require solving several challenges, such as determining the optimal arrangement of the bubbles, defining the target field and external forces for each bubble, controlling the shape and position of each bubble, and analyzing the tension between bubbles. Addressing these challenges is highly complex. Therefore, further investigation and experimentation are planned to devise solutions to this issue in the future.

## Conclusions

We proposed a shape control method for soap bubbles by adding external forces to a simulation. To control the surface only simulation, a target shape is converted into only an outline region, and the driving forces are computed based on this region. By averaging surface tension across all vertices, we improved the controllability in regions with high curvature. Additionally, we introduced several intermediate shapes that smoothly transition from the initial shape to the target shape, and controlled the magnitude of global oscillations of the soap bubble. Our method can control soap bubbles into various target shapes.

Our control achieves shape changes using the intermediate shapes. However, if the target shape changes faster than the movement of the soap bubble, as in the case treated by Shi and Yu^5^, perfect reproduction is challenging with our control. We will plan to extend our method to control such shapes, as for future works. Additionally, we want to move a bubble position while controlling their shape. In our current method, a position of a target shape is fixed. Therefore, we are planning to develop a method that the target field moves like soap bubbles. Enabling shape control for simulations considering the thickness of soap films also remains a challenge for future research.

## Supplementary Information

Supplementary Information.

## Data Availability

The data generated during the current study is available from the corresponding author on reasonable request.
